# A WUSCHEL-Independent Stem Cell Specification Pathway Is Repressed by PHB, PHV and CNA in Arabidopsis

**DOI:** 10.1371/journal.pone.0126006

**Published:** 2015-05-26

**Authors:** Chunghee Lee, Steven E. Clark

**Affiliations:** Department of Molecular, Cellular and Developmental Biology, University of Michigan, Ann Arbor, Michigan, United States of America; Universidad Miguel Hernández de Elche, SPAIN

## Abstract

The homeostatic maintenance of stem cells that carry out continuous organogenesis at the shoot meristem is crucial for plant development. Key known factors act to signal between the stem cells and an underlying group of cells thought to act as the stem cell niche. In *Arabidopsis thaliana* the homeodomain transcription factor WUSCHEL (WUS) is essential for stem cell initiation and maintenance at shoot and flower meristems. Recent data suggest that the WUS protein may move from the niche cells directly into the stem cells to maintain stem cell identity. Here we provide evidence for a second, previously unknown, pathway for stem cell specification at shoot and flower meristems that bypasses the requirement for WUS. We demonstrate that this novel stem cell specification pathway is normally repressed by the activity of the HD-zip III transcription factors PHABULOSA (PHB), PHAVOLUTA (PHV) and CORONA (CNA). When de-repressed, this second stem cell pathway leads to an accumulation of stem cells and an enlargement of the stem cell niche. When de-repressed in a *wus* mutant background, this second stem cell pathway leads to functional meristems with largely normal cell layering and meristem morphology, activation of *WUS* cis regulatory elements, and extensive, but not indeterminate, organogenesis. Thus, WUS is largely dispensable for stem cell specification and meristem function, suggesting a set of key stem cell specification factors, competitively regulated by WUS and PHB/PHV/CNA, remain unidentified.

## Introduction

Stem cells have a crucial role as undifferentiated cells that perpetuate themselves and give rise to differentiating daughter cells. Plants form various types of stem cell populations throughout their lifespan, including shoot, root and flower meristems. Above-ground organs are derived from shoot meristems [1, 2].

The shoot meristem contains centrally-located stem cells surrounded by peripheral daughter cells that make a switch toward organ formation and eventual differentiation. In addition to lateral organs, the shoot meristem also gives rise to lateral shoot meristems and lateral flower meristems [1, 2].

The key to shoot meristem function is the homeostatic maintenance of stem cells, while allowing appropriately-positioned daughter cells to begin to differentiate, forming new organs and tissues. The fate of dividing stem cells are determined by position, with central- and apical-positioned daughters remaining stem cells, while other daughters switch towards differentiation [3, 4].

A key factor regulating stem cell specification in plants is the WUSCHEL (WUS) homeodomain-containing transcription factor. *WUS* is expressed in the Organizing Center (OC), which comprises the niche cells immediately basal to the shoot and flower stem cells. *WUS* expression in the OC specifies overlying cells as stem cells in a non-cell autonomous manner [5, 6]. *wus* mutants lack identifiable shoot and flower meristems [5]. One of the best-studied pathways regulating *WUS* transcription is the CLV signaling pathway. CLV signaling restricts *WUS* expression to the basal daughter of the L3 stem cells, thus limiting the size of the OC and hence, the size of the stem cell population [7, 8]. Many components of CLV signaling have been identified [6, 9–15]. *CLV3*, whose expression appears to mark the stem cell population, codes for the precursor of the peptide ligand that binds to CLV receptor complexes in the L3 cell layer [11, 12, 16–19]. Mutations in the CLV genes result in the opposite phenotype to *wus*, namely enlarged stem cell populations leading to increased organ number and meristem size, all resulting from expanded WUS expression [5, 7, 10, 20–22]. CLV receptor activation represses the activity of the lipid-modified and lipid-binding protein phosphatases POL and PLL1, which are required for *WUS* expression maintenance [13, 15, 23]. While CLV3 signaling limits *WUS* expression, WUS protein promotes *CLV3* transcription. Recently, it has been shown that WUS protein, expressed in the niche, moves though plasmodesmata into the overlying stem cells and this movement is required for WUS function and stem cell activity as well as it binds directly to CLV cis elements [24, 25].

In loss-of-function *wus* mutants, seedlings lack a functional shoot meristem, leading to a differentiated apex [5]. Later, through an unknown pathway, *wus* mutants form adventitious shoots from tissue in between the cotyledons that establish one-to-several leaves. These lateral shoots do not form meristem-like structures, so the mechanism of leaf formation is unknown. The process continues reiteratively in *wus* mutants, with subsequent adventitious shoots forming in the axils of existing leaves. Eventually, *wus* mutants make a type of floral transition, with the adventitious shoots occasionally forming a flower primordium instead of a leaf. These flowers also lack meristem activity, lacking nearly all whorl 3 and 4 organs. In addition to the necessity of *WUS* for stem cell establishment and maintenance, *WUS* over-expression leads to ectopic stem cells within the shoot and flower meristem [7, 8, 26, 27], and in many locations in combination with *SHOOTMERISTEMLESS* (*STM*) over-expression [26, 27]. This suggests that WUS is, in combination with STM, the key player in stem cell specification and initiation of the shoot meristem program. Indeed, reviews of the literature place WUS as the central node of meristem specification and homeostatic control [28, 29].

Genetic studies have led to the identification of many other factors affecting stem cell specification and maintenance. Consistent with the proposed central role for WUS in stem cell maintenance, those factors when analyzed in detail converge to either regulate the *WUS* expression domain or the organ specification pathway represented by *STM* [28–32].

Despite this significant evidence for the centrality of WUS in stem cell specification, some prior studies hinted at a separate WUS-independent stem cell pathway. (1) The *CLV3*::*GUS* reporter, which is typically associated with stem cell identity, is weakly and diffusely expressed in some apical structures of *wus* mutants [8]. (2) *WUS* mRNA expression in the inflorescence shoot apical meristem is low compared to expression at embryonic, seedling, flower or lateral shoot meristems [7, 33]. (3) In portions of massively enlarged *clv* shoot apical meristems, *WUS* expression is lost (suggesting stem cell maintenance in the absence of *WUS*) [33]. However, the inflorescence shoot apical meristem of strong *clv* mutants are very large, derived, and abnormal, making firm conclusions difficult. (4) Some weak suppression of the *wus* phenotype may have been observed in a combination with the heterozygous *men1* activation-tagged allele of miR166g [34]. However, no meristem has been observed in the absence of WUS, leaving this an open question.

Even with extensive efforts described to identify stem cell regulators, major gaps in understanding stem cell control remain. In an effort to identify other factors controlling stem cell specification, we conducted a mutagenesis of *pol-6* single mutants, screening for modifiers that would lead to loss of stem cells specifically in a *pol* mutant background. In course of analyzing a novel allele that arose from the screen, we have identified a WUS-independent pathway for stem cell specification, meristem development and organogenesis.

## Results

### Identification of a novel *AGO10* allele

To identify novel components of stem cell homeostatic control, we performed ethyl methanesulfnate (EMS) mutagenesis on *pol-6* seeds. Seeds were collected in pools from 884 M1 individuals and the M2 population was screened for shoot and flower meristem termination. Putative modifier mutants were crossed to wild-type L*er* to test if the meristem termination phenotype was dependent on the presence of the *pol* mutation. Isolate #361 displayed seedling shoot meristem termination that was largely dependent on the presence of the *pol* mutation. In the wild-type L*er* outcross for isolate #361, 22 of 369 plants displayed meristem defects, and 19 of these were homozygous for *pol-6*.

**Fig 1:**
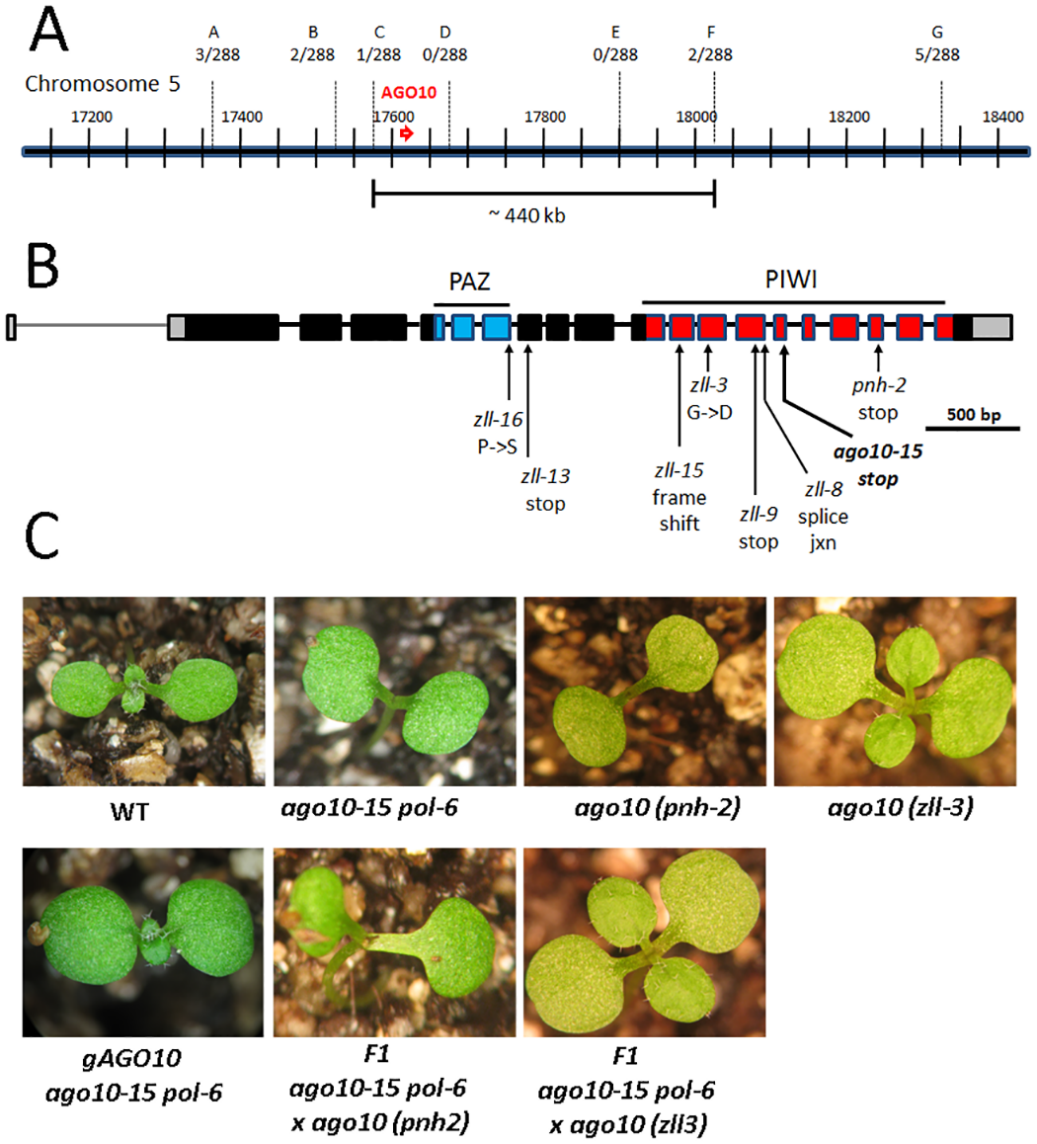
Identification of a novel *ago10* allele. (A) Map-based cloning of the isolate #361 *pol* modifier. Letters indicate the location of fine mapping markers (see Table A in S1 File) and the number of recombinants among 288 mapping chromosomes. The 440 kb region delimited by the closest recombinants is shown. (B) Diagram of the *AGO10* genomic organization. The location of the coding sequences for the PAZ and PIWI domain as well as the locations and nature of various *ago10* alleles are indicated. (C) Seedlings phenotypes of wild-type (WT), *ago10-15 pol-6* double mutant, *ago10* ^*phn-2*^ and *ago10* ^*zll-3*^ alleles are shown. Complementation of *ago10-15 pol-6* with the *AGO10* genomic DNA as well as allelism tests with the *ago10* ^*phn-2*^ and *ago10* ^*zll-3*^ alleles are shown.

The F2 population of the L*er* cross was used for map-based cloning of the modifier mutant. Among a population of 3888 F2 plants, 144 enhanced mutants were identified and DNA was collected for analysis. Using SSRP, CAPS, and dCAPS markers, the modifier mutation was localized to a 440 kb region on chromosome 5 (Fig 1A). Genes in the region were sequenced. In the *ARGONAUTE10* (*AGO10*) gene, also known as *ZWILLE*/*PINHEAD*, a G to A transition mutation in the 14^th^ exon was identified, resulting in a nonsense mutation (Fig 1B). We numbered the new allele *ago10-15* following the nomenclature from a recent publication [35]. To confirm the lesion within *AGO10* was responsible for the meristem termination in the original isolate, we transformed *AGO10* genomic DNA [35] into *ago10-15 pol-6* plants and observed rescue of the meristem termination phenotype (Fig 1C). In addition, among the F1 progeny of our *ago10-15 pol* isolate crossed to *ago10* ^*zll-3*^ and *ago10* ^*pnh-2*^ [36, 37] we observed meristem termination and an increase in the mean number of carpels per flower typical of *ago10* mutants, with post-embryonic growth dependent on adventitious meristems as typically observed in *ago10* mutants (Fig 1C, Fig A in S1 File).

**Fig 2:**
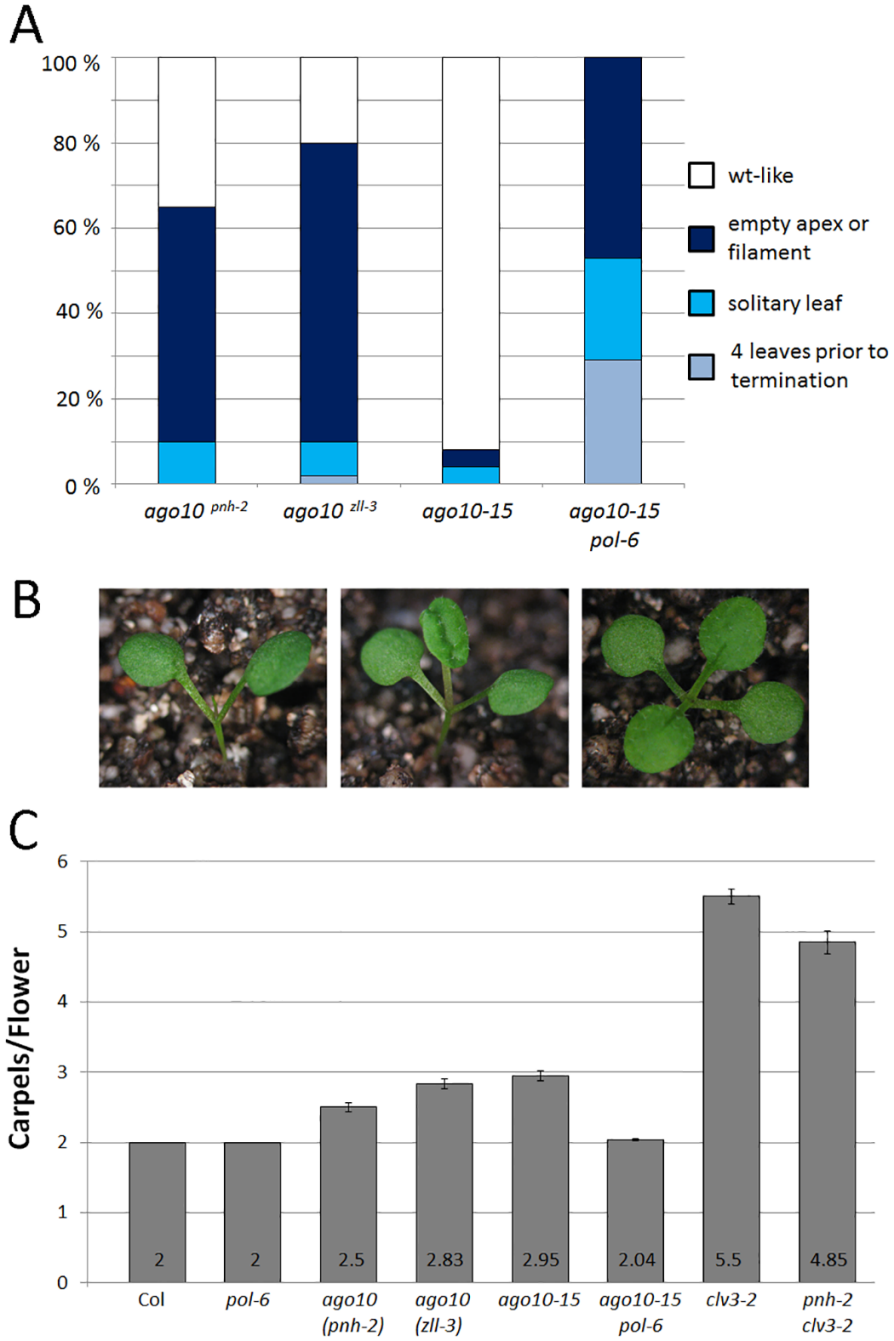
The *ago10-15* allele. (A) Frequency of seedling phenotypes for *ago10* alleles. Samples sizes as follows: *phn-2* (69), *zll-3* (54), *ago10-15* (23), *ago10-15 pol-6* (100). (B) Representative phenotypes for *ago10-15 pol* with apex filament, solitary leaf and four leaves prior to termination are shown. (C) Mean number of carpels per flower with standard error of the mean for wild-type and various mutants are shown.

Interestingly, the *ago10-15* allele we isolated differs from most published *ago10* alleles in *ago10-15* meristem termination appeared with 10% penetrance, while published alleles typically have greater than 60% penetrance (Fig 2A) [36, 37]. This is despite the severe effect *ago10-15* should have on AGO10 protein and the fact that the enlargement observed in *ago10* flower meristems is as strong in *ago10-15* as in other alleles (Fig 1B). *ago10* ^*pnh-4*^ and *ago10* ^*pnh-11*^ also have weak penetrance of the seedling meristem termination, but the DNA lesions in these alleles have not been described [37].

*ago10-15 pol-6* seedlings displayed completely penetrant meristem termination, characterized by a differentiated flat apex, a filament, a solitary leaf, or at most the production of up to four leaves followed by termination (Fig 2A and 2B). Given that neither *pol-6* nor *ago10-15* exhibit significant meristem termination, this represents a synergistic interaction. A different genetic interaction was observed post-embryonically, where *AGO10* fulfills a very different role; namely, limiting meristem development [36]. Presumably because of these antagonistic roles of *AGO10* in the embryonic shoot meristem compared to the postembryonic shoot/flower meristems, *pol-6* suppressed the flower meristem enlargement of *ago10-15* (Fig 2C).

To further examine the *CLV*/*AGO10* genetic interactions, we generated double mutants between the previously characterized *ago10* ^*pnh-2*^ and *clv3-2* [22, 36, 37]. In these double mutants there was no enhancement of the shoot or flower meristem enlargement compared to *clv3-2* alone, with flowers either similar to *clv3-2* flowers or occasionally replaced by filamentous structures (Fig 2C).

### PHB/PHV/CNA act independently of the CLV pathway

The lack of internal consistency of genetic interactions between *ago10* mutants and CLV pathway mutants makes a clear conclusion difficult. This variable genetic response for *ago10* likely reflects the overlapping and antagonistic function of known AGO10 targets. Unlike AGO1, which has a broad ranging function in miRNA action [38–42], AGO10 appears to be fairly specific for the miR165/166 family, where AGO10 paradoxically prevents these miRNAs from repressing their targets [35, 43–45]. Genetic alteration of miR165/166 function results in meristem defects [34, 43]. In turn, the miR165/166 targets, the homeodomain-leucine zipper class III (HD-zip III) genes, have complicated overlapping and antagonistic roles in many aspects of meristem development [46–49]. Critically, *REVOLUTA* (*REV*) plays an important role in promoting shoot and flower meristem initiation, while *PHABULOSA* (*PHB*), *PHAVOLUTA* (*PHV*) and *CORONA* (*CNA*) play redundant, antagonistic roles in limiting meristem development [48, 49]. *rev* and *phb*/*phv*/*cna* mutants suppress each other’s meristem phenotypes post-embryonically [49]. Thus, *ago10* mutants appear to have both a reduction of *REV* activity, leading to meristem loss, and a reduction on *PHB*/*PHV*/*CRN* activity, leading to meristem enlargement. Because these antagonistic relationships might mask critical meristem functions, we explored the interactions of *PHB*/*PHV*/*CNA* with the CLV/WUS pathway. *phb phv cna* triple mutants have been only briefly described as exhibiting inflorescence and flower reminiscent of *clv* mutants [49], but the relationship between *PHB*/*PHV*/*CNA* and other meristem regulators has remained unexplored.

**Fig 3:**
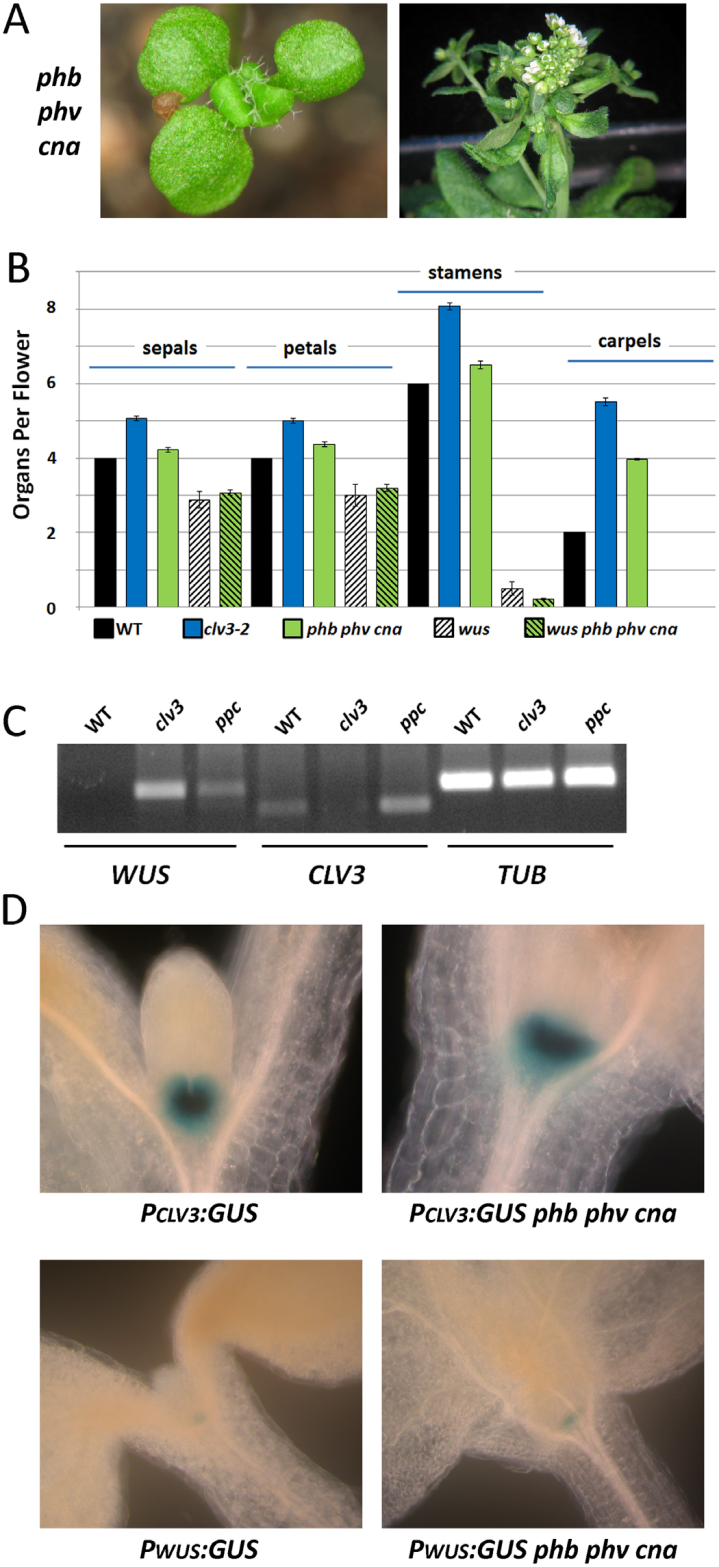
PHB PHV and CNA restrict meristem size. (A) *phb phv cna* triple mutants displaying tricotyledon phenotype (left) and inflorescence stem fasciation (right) are shown. (B) Mean numbers of floral organs per flower with standard error of the mean for wild-type and various mutants are shown. (C) Semi-quantitative RT-PCR measuring the accumulation of *WUS*, *CLV3* and control *TUBULIN* transcripts in wild-type (WT), *clv3-2* and *phb phv cna* (ppc) 5 days-old seedlings. (D) A comparison of *PCLV3*:*GUS* and *PWUS*:*GUS* reporter line activity of wild-type (left) and *phb phv cna* (right) seedlings.

**Fig 4:**
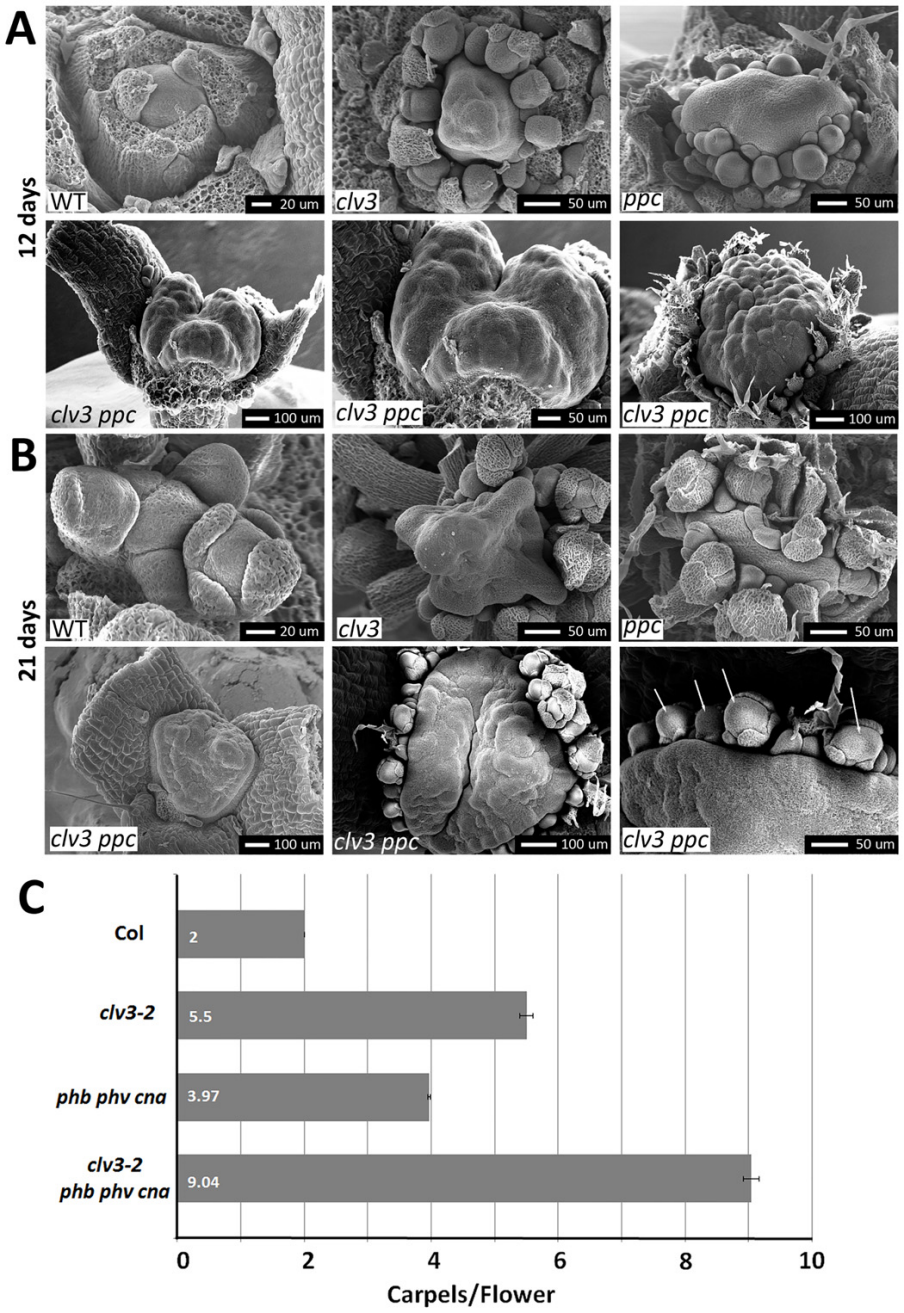
*clv3* enhances *phb phv cna* meristem defects. (A) Shoot apical meristems of 12 day old wild-type (WT), *clv3-2*, *phb phv cna*, and *clv3 phb phv cna* mutants imaged by scanning electron microscopy. Note the fasciated meristem of *phb phv cna*. *clv3 phb phv cna* mutants occasionally lack all organ primordia as shown the bottom left panel and at higher magnification in bottom center panel. cot, cotyledon. (B) Shoot apical meristems of 21 days-old plants. Note the continued lack of organ primordia in the bottom left panel. Bottom center panel enlarged on bottom right to show enlarged flower meristems (arrows). (C) Mean number of carpels per flower with standard error of the mean for various genotypes.

*phb phv cna* mutants, using the null alleles *phb-13*, *phv-11*, and *cna-2*, often displayed a tricotyledon phenotype, stem fasciation, and silique enlargement (Fig 3A). Similar to *clv* mutants, *phb phv cna* mutants developed an increase in the number of flower organs in each whorl, with the largest deviation from wild-type found in the central carpels (Fig 3B). When analyzed by scanning electron microscopy (SEM), the shoot apical meristems of *phb phv cna* mutants were enlarged and misshapen compared to wild-type, with fasciation (the conversion of the meristem into a line) often observed (Fig 4A and 4B).

To test the effect of *phb phv cna* on expression of the key markers/regulators *CLV3* and *WUS*, we first measured total transcripts by semi-quantitative RT-PCR in wild-type, *phb phv cna* and *clv3-2* seedlings. We observed an up-regulation of both *CLV3* and *WUS* transcript accumulation in *phb phv cna*, consistent with the increase in meristem size in these mutants (Fig 3C). We then crossed previously characterized *PWUS*:*GUS* and *PCLV3*:*GUS* reporters into the *phb phv cna* mutant background [26, 50]. The expression of both reporters were expanded laterally in 5 days-old *phb phv cna* seedlings compared to wild-type (Fig 3D), suggesting that PHB/PHV/CNA may act in the CLV/WUS pathway to limit *WUS* expression.

**Fig 5:**
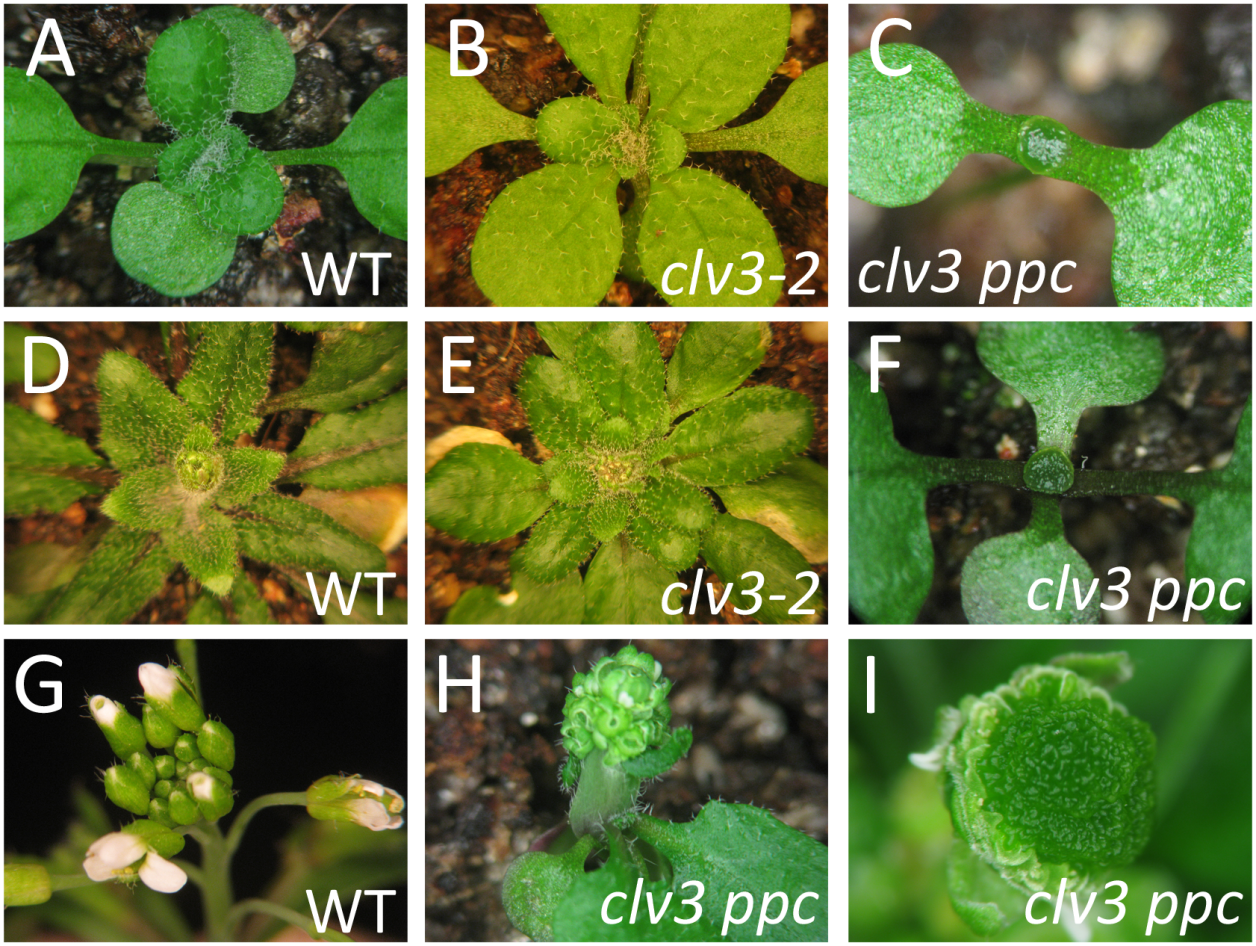
Phenotypes of *clv3-2 phb phv cna* plants. Shown are wild-type (A,D), *clv3-2* (B,E) and *clv3-2 phb phv cna* (C,F) plants at 15 days-old (A-C) and 26 days-old (D-F). Note the massive meristems and lack of organ primordia for *clv3-2 phb phv cna*. Wild-type (G) and *clv3-2 phb phv cna* (H) inflorescences are shown. *clv3-2 phb phv cna* flowers (I) continue proliferation and massive accumulation of stem cells.

To test whether PHB/PHV/CNA act within the CLV/WUS pathway, we crossed *phb phv cna* to the strongest *clv* mutant allele *clv3-2*, which is epistatic to other mutations in other *CLV* genes [10, 22]. Surprisingly, the morphology of *clv3 phb phv cna* plants was dramatically altered and enhanced compared to *clv3-2* single mutants. At very early stages of vegetative development, massively enlarged stem cell populations accumulated, often without any evidence of lateral organ formation (Fig 5A–5C). *clv3 phb phv cna* quadruple mutants all eventually initiated organ primordia while the shoot meristem continued to accumulate additional stem cells (Fig 5D–5F). Inflorescence bolting was impaired in *clv3 phb phv cna* plants, presumably due to the massive size of the meristem (Fig 5G and 5H).

*clv3 phb phv cna* flower meristems were also massively enlarged compared to *clv3* single mutants, as apparent from flower morphology, SEM analysis and organs produced (Figs 4B, 4C and 5I). *clv3 phb phv cna* flowers averaged over 9 carpels per flower and exhibited indeterminate stem cell proliferation leading to massive populations of meristem-like cells even very late into flower development. *clv3 phb phv cna* flowers were completely sterile.

**Fig 6:**
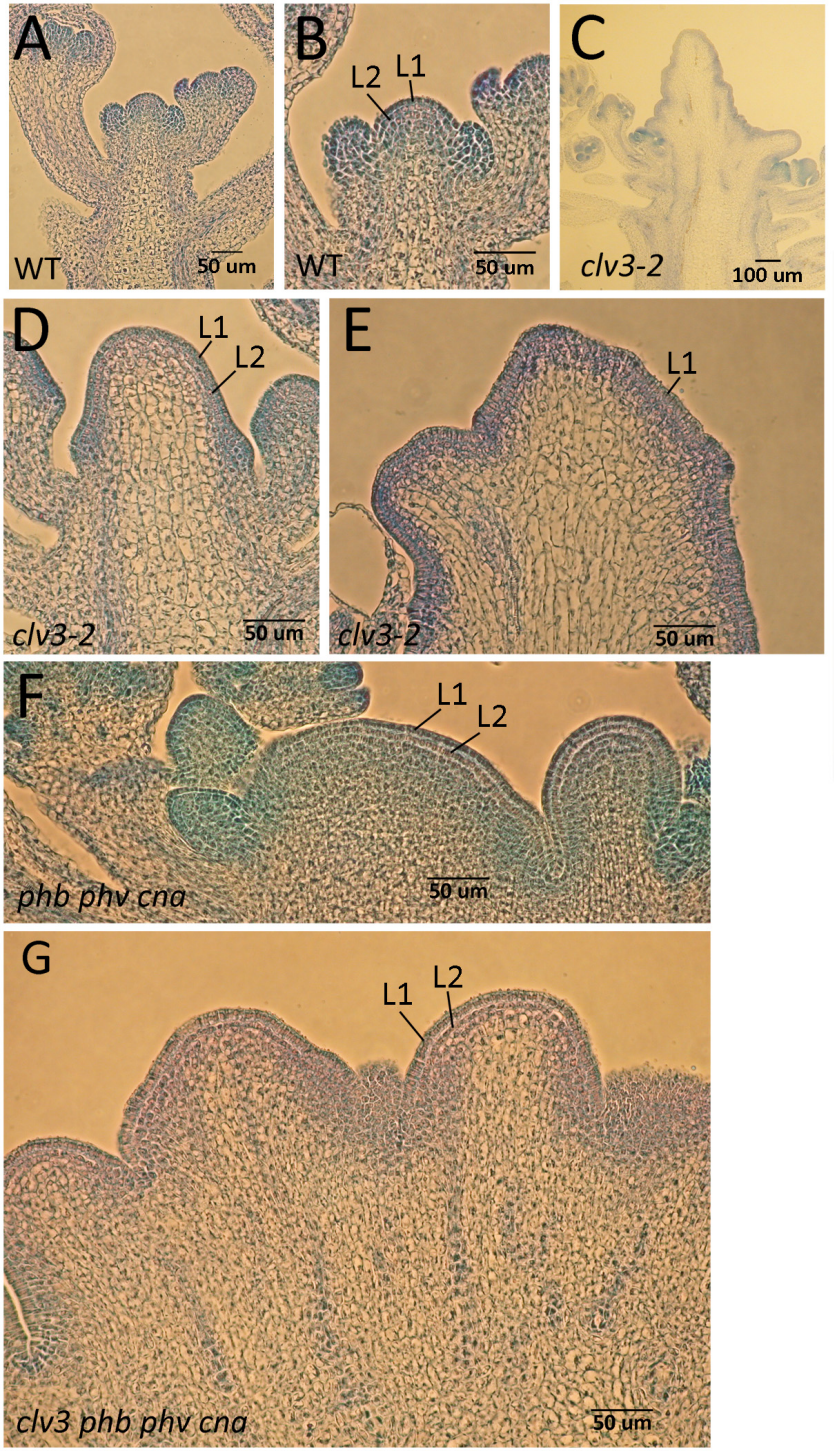
Stem cell layering maintained in *phb phv cna*. Histological sections of wild-type (A, B), *clv3-2* (C-E), *phb phv cna* (F) and *clv3-2 phb phv cna* (G) inflorescence shoot apical meristems. Histologically identifiable L1 and L2 layers are labeled. Note breakdown in clear layering in central apex of *clv3-2* in (D) and the columnar shape of the L1 layer cells in (E) and (G). *phb phv cna* mutants maintain clear layering pattern even in enlarged meristems (F).

Wild-type shoot apical meristems have two very clear clonal cell layers (L1, L2) that undergo strictly anticlinal divisions [3, 51–53]. The underlying L3 layer is smaller and undergoes divisions in both anticlinal and periclinal orientations. *clv* mutants appear to lose the L3 layer as the OC-factor *WUS* expands into the L3 cells [7]. This affected the morphology of the *clv* meristems, with a lack of a clear L3 layer and a breakdown of all cell layering in the center of the larger *clv* meristems (Fig 6D and 6E). In addition, L1 cells in *clv* meristems often adopted a more columnar shape (Fig 6E). *phb phv cna* mutants, despite a similarity to *clv* mutants, retain a clear layering patterning that includes an L3 layer (Fig 6F). In addition, we did not observe columnar L1 cells in *phb phv cna* meristems. The *clv3 phb phv cna* quadruple mutants developed columnar L1 cells and lacked a clear L3 layer similar to *clv* single mutants (Fig 6G).

### PHB/PHV/CNA act independently of WUS

**Fig 7:**
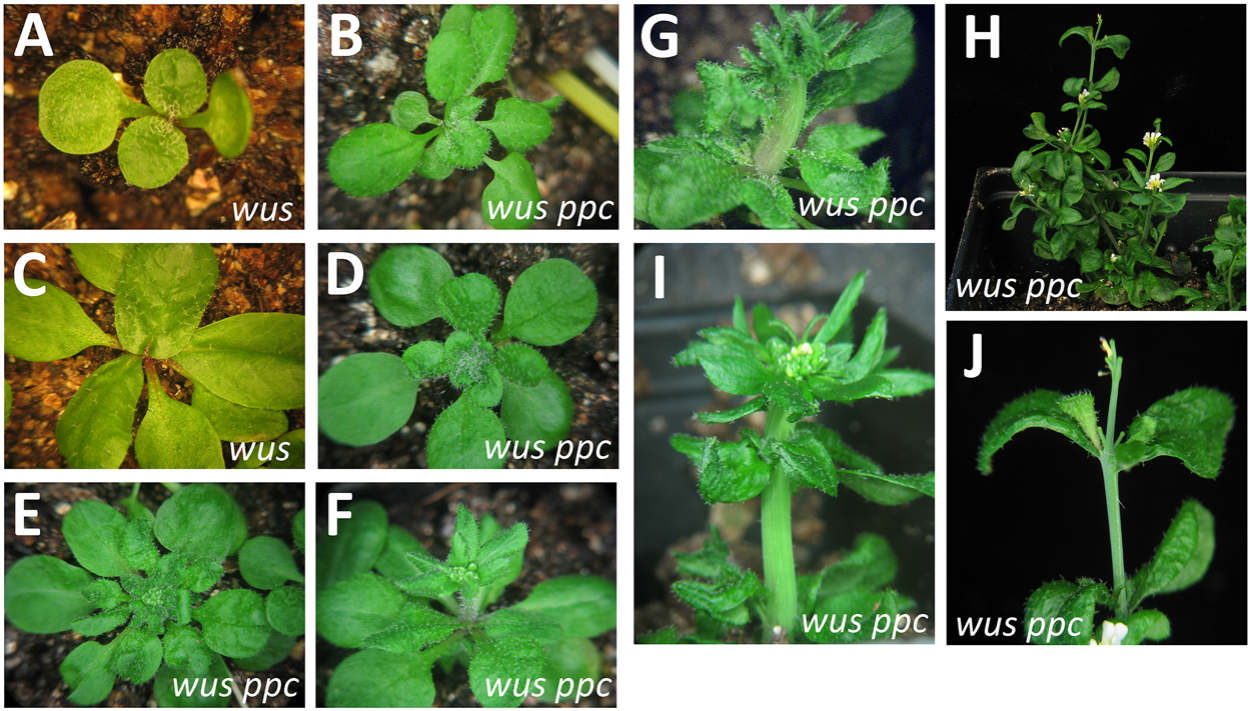
*phb phv cna* suppress the *wus* phenotype. (A, C) *wus-1* single mutants at 18 and 27 days-old. (B, D) *wus phb phv cna* plants with a single adventitious meristem leading to a full rosette at 15 and 26 days-old. (E, F) *wus phb phv cna* plants with emerging inflorescences. (G, I) *wus phb phv cna* plants with fasciated inflorescence stems. (H, J) Late-arising adventitious shoots on *wus phb phv cna* plants resemble *wus* mutants.

Even though PHB/PHV/CNA act independently of the CLV pathway, they could act in a CLV-parallel pathway controlling *WUS* expression or activity. Indeed, most or all stem cell regulators studied in detail to date appear to act through *WUS* [28–30, 32]. To test this idea, we generated the *wus-1 phb phv cna* quadruple mutant. These plants were assayed as progeny of a *wus/+ phb phv cna* parent. *wus* mutants display a fully penetrant differentiation of the embryonic apex, leading to an absence of leaf primordia that is clearly visible within 5–7 days after germination [5]. *wus* mutants subsequently form leaves from presumed adventitious shoots at the apical region of the seedling and later in the axil of existing leaves. No meristems have been identified within the developing post-embryonic structures of *wus* mutants. Surprisingly, *wus phb phv cna* quadruple mutants developed 1–8 leaves before the initial shoot apical meristem termination (mean = 2.8 ± 1.5 (standard deviation), n = 26). After the initial termination of the shoot apical meristem, *wus phb phv cna* mutants rapidly formed a single or multiple adventitious meristems (Fig 7A–7D). These meristems were often capable of developing a full rosette of leaves and an inflorescence with multiple cauline leaves and flowers prior to terminating (Fig 7E and 7F). While no indeterminate structures formed in *wus phb phv cna* plants, the shoots that were present had much of the organogenic capacity typically observed in wild-type shoot meristems. Fasciated stems were occasional observed suggesting an ectopic accumulation of stem cells despite the lack of WUS function (Fig 7G and 7I).

**Fig 8:**
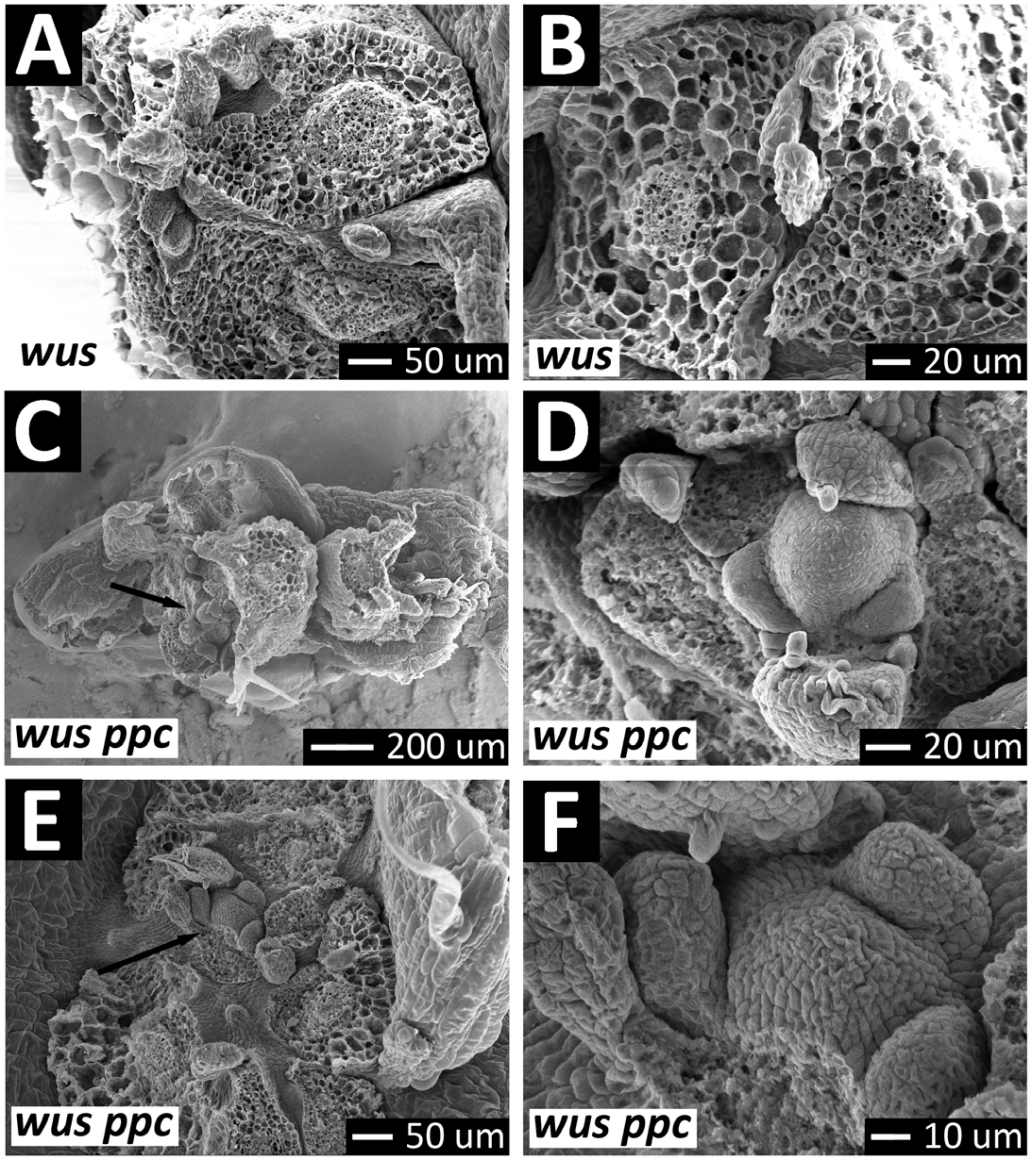
Meristems in *wus phb phv cna* plants. (A, B) Dissected *wus* seedlings imaged by SEM reveal no meristem-like structures. (C-F) Dissected *wus phb phv cna* seedlings reveal meristem-like structures (C,E—arrows). At higher magnification, meristem structures similar to wild-type are observed (D,F; c.f, Fig 4A).

**Fig 9:**
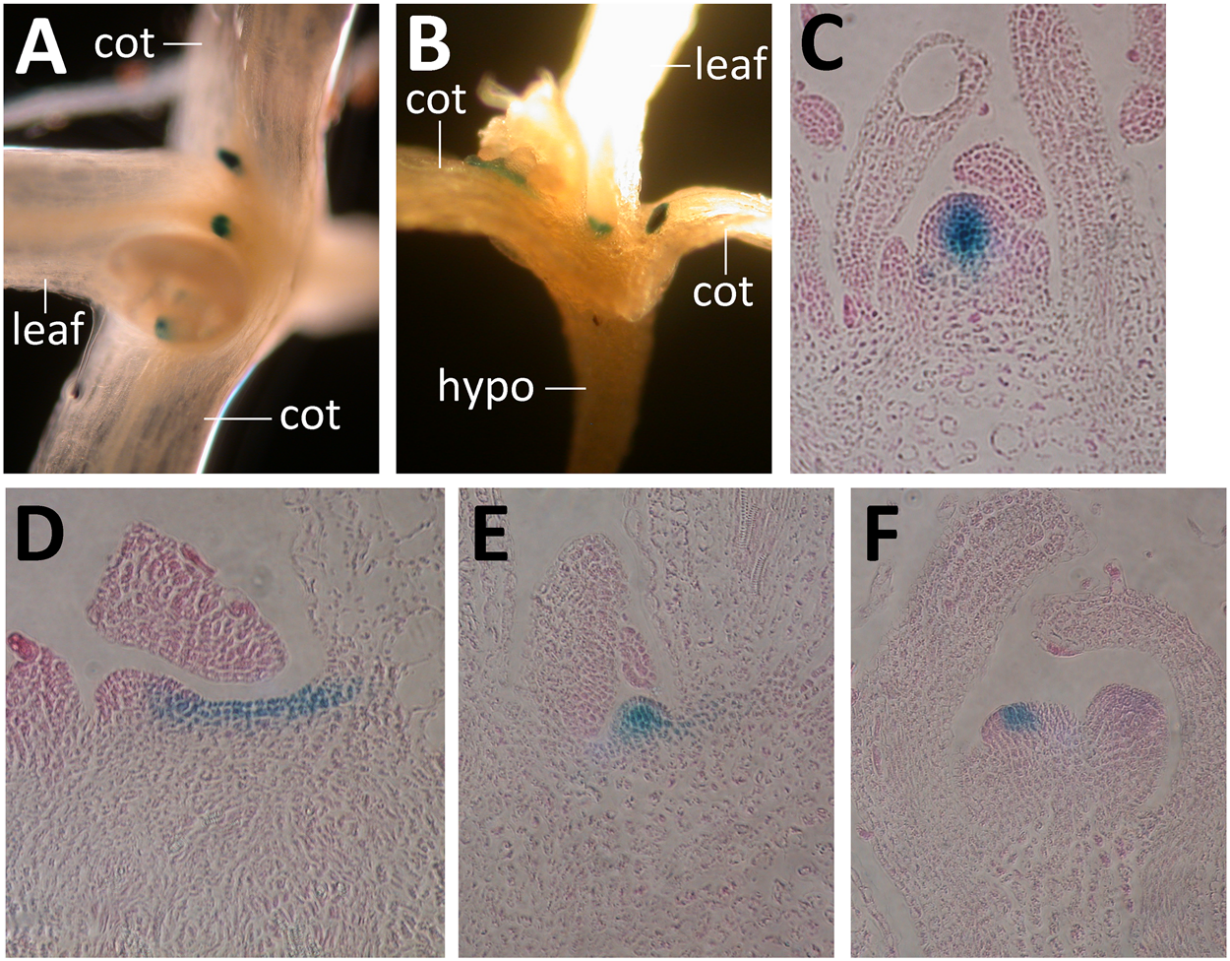
*Pwus*:*GUS* activity in *wus phb phv cna*. (A, B) Top and side views of *wus phb phv cna* plants carrying *PWUS*:*GUS*. Note the reporter activity in the axils of leaves and cotyledons. cot, cotyledon; hypo, hypocotyl. (C-F) GUS-stained and sectioned *PWUS*:*GUS* plants (C) GUS-stained and sectioned *PWUS*:*GUS* wild type 12 day-old seedling and (D-F) *wus phb phv cna* mutant plants at 12 days (D) and 18 days old (E, F), respectively.

**Fig 10:**
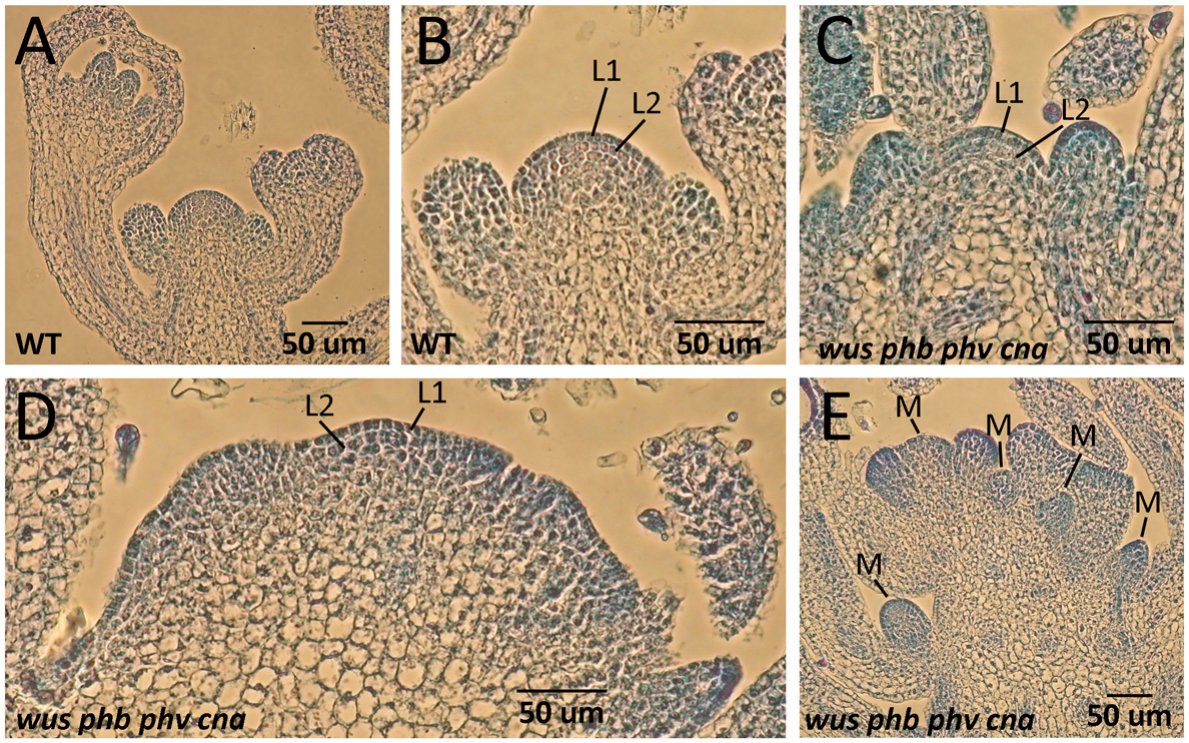
Histology of *wus phb phv cna* meristems. (A, B) Histology of wild-type shoot apical meristems reveals a layering pattern in the central stem cells. (C, D) Histology of *wus phb phv cna* shoot meristems reveals a layering pattern in normally-sized meristems (C) that is less organized in larger meristems (D). (E) Extensive development of lateral or adventitious meristems (M) revealed by sections of *wus phb phv cna* plants.

To determine what structures were responsible for this organogenesis, we analyzed *wus phb phv cna* mutants by SEM. While we never observed any meristem-like structures among *wus* seedlings (Fig 8A and 8B), we readily observed meristem-like structures at the apical region of *wus phb phv cna* plants (Fig 8C–8F). The *wus phb phv cna* meristems exhibited the dome shape typical of wild-type meristems (c.f., Fig 8D, 8F to Fig 4A). Histological sectioning of *wus phb phv cna* mutants revealed that normal-sized meristems retained the L1/L2 layering (Figs 9E, 9F and 10C). *wus phb phv cna* meristems larger than wild-type occasionally exhibited a loss of clear layering (Fig 10D).

The structures formed in *wus phb phv cna* quadruple mutants had both the appearance and function of normal shoot meristems, although they were not fully indeterminate. To determine if they established a normal Organizing Center comparable to wild-type meristems, we assayed *Pwus*:*GUS* activity in the quadruple mutant. This marker line has been previously characterized and showed normal expression in the internal layers of wild-type shoot meristems [6, 26, 54] (Fig 9C). Within *Pwus*:*GUS wus phb phv cna* seedlings, we observed patches of GUS activity in the axils of cotyledons, leaves, and in the apical region—all locations where we detected the adventitious shoot and meristems (Fig 9A and 9B). To determine if these GUS puncta corresponded to meristems, we fixed and sectioned *Pwus*:*GUS wus phb phv cna* plants. Here we observed both GUS activity in a relatively large pool of cells (Fig 9D), and puncta of GUS activity that corresponded to the internal cell layers of apparent meristems (Fig 9E and 9F). In no case did we observe a meristem-like structure in *Pwus*:*GUS wus phb phv cna* plants that lacked GUS activity, suggesting that meristems of *wus phb phv cna* plants have a relatively normal *WUS* activation in internal layers consistent with an OC. Later arising adventitious shoot of the quadruple mutant lacked evidence of meristem activity and were indistinguishable from *wus* single mutants (Fig 7H and 7J).

Within the developing flowers, *wus* appeared largely epistatic to *phb phv cna*. The quadruple mutants never formed the central carpels. The mean numbers of flower organs were similar between *wus phb phv cna* and *wus* single mutants (Fig 3B). Thus, the phenotypic suppression of *wus* by *phb phv cna* was limited to vegetative and early reproductive stages.

During the construction of the *wus phb phv cna* quadruple mutant line, we failed to readily observe *wus* suppression unless all three HD-zip III genes were homozygous mutant. For example, *wus*/*wus phb*/*phb phv*/+ *cna*/*cna* plants were not clearly different from *wus* single mutants (Fig B in S1 File).

*WOX5*, a *WUS* homolog, functions at the root meristem to promote stem cell specification [55, 56]. To determine if *wus* phenotypic suppression in the *phb phv cna* background was the result of up-regulation of *WOX5*, we assessed *WOX5* trascription by semi-quantitation RT-PCR in wild-type, *wus phb phv cna*, and *clv3 phb phv cna*. Using two different primer sets, no differences in *WOX5* transcript accumulation were apparent (Fig C in S1 File).

## Discussion

### A WUS-independent stem cell pathway

Previous research had indicated that WUS is critically required for stem cell initiation and maintenance at shoot and flower meristems [5–8, 26, 27]. Here we provide clear evidence that WUS is dispensable for a functional shoot meristem. *wus phb phv cna* quadruple mutants showed evidence of both embryonic shoot meristem function (in the form of post-embryonic leaf development) and the formation of functional shoot meristems post-embryonically. These post-embryonic meristems in the absence of WUS had many features of normal meristem morphology, histology and activation of the key OC marker (i.e., *WUS* cis elements). While the post-embryonic vegetative shoot meristems of *wus phb phv cna* plants were not indeterminate, they were capable of extensive organogenesis consisting of a full rosette of vegetative leaves, cauline leaves and over a dozen flower primordia. We even observed fasciated stems and meristems in the quadruple mutant, suggesting ectopic stem cell accumulation in the absence of WUS.

That PHB/PHV/CNA act in a WUS-independent pathway to control stem cell initiation and function is consistent with genetic interactions with *clv3-2*, where we observed a strong enhancement of stem cell accumulation and loss of organogenesis in the *clv3-2 phb phv cna* quadruple mutant. The resulting plants were severely abnormal, often lacked the ability to form lateral organs, and accumulated massive populations of stem cells. *clv3-2 phb phv cna* flowers were similarly enhanced compared to *clv3-2* alone, with significant increases in organ number, stem cell accumulation and indeterminate growth. Taken together, we conclude that PHB/PHV/CNA represent a pathway parallel to CLV/WUS for stem cell initiation and maintenance, and that the role of PHB/PHV/CNA in this pathway is to limit the stem cell population.

The failure to clearly identify AGO10, miR165/166 and the HD-zip III genes as representing a CLV/WUS-independent stem cell pathway despite intense study likely rests on two features of these regulators [34–36, 43, 49]. First, REV antagonizes PHB/PHV/CNA function in post-embryonic meristem development. Thus, genetic manipulation of AGO10 or miRNA165/166 function would have a mixed effect by simultaneously promoting and inhibiting stem cell specification. Thus, *wus* was described as epistatic to miR166 over-expression caused by the *jabba1-D* allele [43] and to *ago10-12* [35], while *ago10 clv3* double mutants failed to display any meristem enhancement (Fig 2). The second feature likely masking a CLV/WUS-independent pathway is the apparent genetic requirement to completely eliminate PHB/PHV/CNA activity in order to observe *wus* suppression. Even a single wild-type allele of PHB/PHV/CNA appeared to prevent *wus* suppression (Fig B in S1 File). This suggests that even very limited PHB/PHV/CNA activity can block stem cell initiation in a *wus* background.

### The PHB/PHV/CNA stem cell pathway

**Fig 11:**
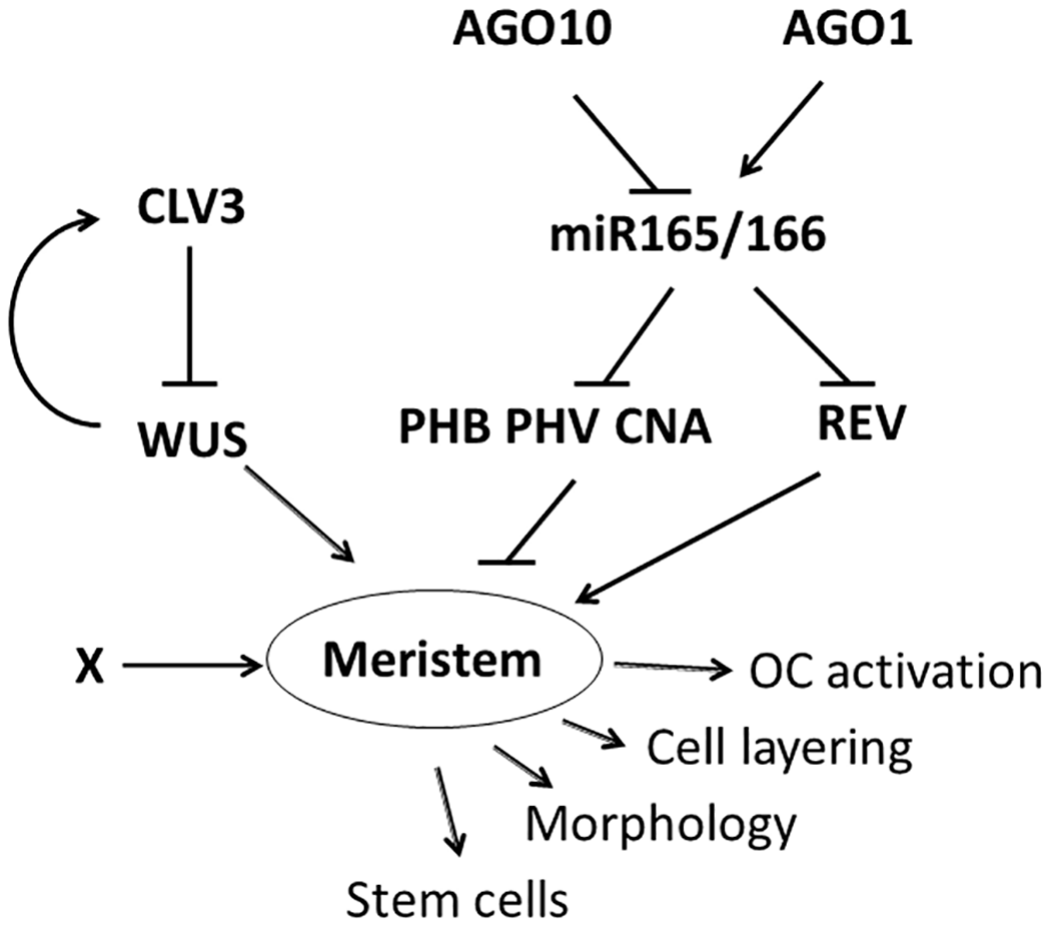
A model for stem cell control. A model for regulatory control of stem cells at the shoot meristem is shown. We propose WUS and PHB/PHV/CNA antagonistically control meristem development. Speculation that either REV or an unknown factor (X) drives meristem development in the absence of WUS and PHB/PHV/CNA is also shown.

If PHB/PHV/CNA act in a WUS-independent pathway, how do these genes control stem cell specification? From the phenotypes and genetic interactions, it is clear that PHB/PHV/CNA act to limit the stem cell population post-embryonically. This is in contrast to their role in promoting embryonic shoot meristem formation [48, 49]. PHB/PHV/CNA presumably repress a factor(s) that is capable of activating stem cell initiation and maintenance. Thus, the entire shoot meristem control pathway is antagonistically controlled by WUS and PHB/PHV/CNA (Fig 11). Based on this model, *wus* mutants lack stem cells because nothing balances the meristem repression of PHB/PHV/CNA, while *phb phv cna* mutants accumulate stem cells because of a lack of antagonism for WUS activity. In the absence of both WUS and PHB/PHV/CNA, the shoot meristem pathway is active but poorly regulated, leading to both enlarged and terminated meristems.

This raises the question: what are the genes responsible for shoot meristem pathway? While significant steps have been made in transcriptional profiling of the meristem and screens for WUS direct and indirect targets, we still know little about how WUS executes stem cell identity [57–59]. As WUS targets become further characterized, these would make candidate for antagonistic regulation by PHB/PHV/CNA. One very recently identified WUS target, HECATE1, promotes stems cell specification [60]. This gene could be responsible for the WUS-independent stem cell specification identified in this study. Another interesting candidate for shoot meristem promotion in the absence of WUS is REVOLUTA (REV). REV is antagonized by PHB/PHV/CNA post-embryonically and REV is required post-embryonically for lateral shoot and flower meristem specification [49, 61]. Critically, REV is required for adventitious shoot formation in *wus* mutants, indicating it retains some function in a *wus* background [61]. Directly testing the role of REV in the absence of PHB/PHV/CNA is complicated by the fact that *rev phb phv cna* plants are seedling lethal with the apical portion transformed into a single radically-symmetric cotyledon.

## Material and Methods

### Plant growth and genetic analysis

Arabidopsis seeds were sown on a 2:1:1 mixture of top soil:perlite:vermiculite supplemented with fertilizer and imbibed for 7 days at 4°C. Plants were grown under continuous cool-white fluorescent lights at 22°C. Plants in petri dishes were grown on half-strength Murashige and Skoog salts (Sigma) with 0.8% (w/v) phytoagar. Seeds were imbibed for 4 days at 4°C and grown under continuous cool-white fluorescent lights at 22°C.

*zll-3* and *pnh-2* seeds were obtained from the Arabidopsis Biological Resource Center (ABRC, Ohio State University, Columbus, OH, USA). The *ago10-15* allele was generated from 0.35%-0.40% ethyl methanesulfnate (EMS) mutagenesis on *pol-6* single mutant seeds. EMS mutagenesis screening was performed as described [62]. Approximately 578 M1 plants from 0.35% EMS treated and 306 M1 plants from 0.40% EMS treated were collected as pools of 10 individual plants and 64 M2 seeds from each pool were sown and observed for meristem termination. Putative *pol-6* enhancers showing defective meristem phenotypes were crossed to L*er* to test if the putative enhancer was dominant/recessive, to test if it was dependent on *pol* mutant background.

For measurements of floral organ numbers, 100 flowers were assayed with the following exceptions: Col (30), *pol-6* (50), *pol-6 er* (50), *ago10-15* (120), *ago10-15 pol-6* (140), *clv3-2* (86), *wus* (8).

### Map-based Cloning

The F2 population of isolate #361 x L*er* was used for mapping and cloning the modifier mutant. Among a population of 3888 F2 plants, 144 mutants were identified and DNA was extracted. The primers for the SSLP and CAPS markers used for the fine mapping are shown in Fig 1A and listed in Table A in S1 File. While a few of the fine mapping markers were from prior studies, the majority were designed by using the Monsanto Arabidopsis Polymorphism Sequence Collection Database. All restriction enzymes used were obtained from New England Biolabs or Promega and PCR amplification was done using GoTaq (Promega, USA).

### Complementation of *ago10-15 pol-6*

For the complementation of the *ago10-15 pol-6* double mutants, the pPZP211 vector containing *AGO10* genomic DNA was kindly provided by Xuemei Chen (University of California, Riverside). The provided vector was sequenced and confirmed to include ~2 kb upstream sequence, 4.6 kb *AGO10* genomic coding sequence, and 380 bp terminator sequences. The construct was transformed into the *Agrobacterium* strain GV3101, which was then used to transform Arabidopsis Col and *ago10-15 pol-6* as described [63]. Transgenic plants were isolated by selection on plates containing 50 μg/ml spectinomysin.

### Histological Analysis

Tissue fixation and section were performed as described previously [64]. Eight micrometer sections were prepared using the Leica RM 2065 microtome, stained in 0.025% toluidine blue, and examined with a Nikon OPTIPHOT-2 microscope.

### SEM

SEM analysis was performed as described [65]. Briefly, tissue samples were fixed in 4% gluteraldehyde in a sodium phosphate buffer at 4°C overnight then stained with 0.5% osmium for several days at 4°C. The tissue was then taken through an ethanol dehydration series and critical point dried before mounting with silver paste and gold coating. Images were collected using a Hitachi 3200N SEM.

### GUS staining

*Pwus*:*GUS* and *PCLV3*:*GUS* reporter genes [26, 50] were introduced into the *phb phv cna* mutant by crosses. In the F2 population, *phb phv cna*-like plants were isolated and genotyped using *phb*, *phv*, and *cna* primers in [49]. The presence of the reporter gene was tested by PCR and GUS staining. *Pwus*:*GUS phb phv cna* was crossed to *wus*/+ *phb phv cna* to obtain *Pwus*:*GUS wus phb phv cna*. GUS staining was performed described [14]. The tissue was then fixed, cleared, and embedded in paraffin. Sections (8 μm) were cut from the embedded tissue.

### RT-PCR

5 days-old seedlings of Col, *phb phv cna* and *clv3-2* were used for RNA extraction per manufacturer’s instructions (RNeasy Plant Kit, Qiagen, Alameda, CA) and DNaseI treated using the RQ1 RNase-Free DNase (Promega, USA). Two micrograms of RNA was then reverse-transcrbied using Superscript III Reverse Transcriptase using oligo(dT) primers. The set of primers used are listed in Table B in S1 File.

### Image Analysis

Images were collected with a Zeiss stemi sv11 microscope and captured with a Canon digital camera PowerShot S51S. Images were collated in Photoshop with occasional adjustments to brightness and contrast.

## Supporting Information

S1 File **Table A.** Markers used for map-based cloning of *ago10-15* from Fig 1A.**Table B**. Primers used for *WUS* and *CLV3* transcript analysis. **Fig A.** *ago10-15* allelism test. **Fig B.** *wus* suppression requires *phb*, *phv* and *cna* homozygousity. **Fig C.** *WOX5* transcript accumulation unchanged in *phb phv cna* mutants.
(DOCX)
