# Supplementary material for: A WUSCHEL-Independent Stem Cell Specification Pathway Is Repressed by PHB, PHV and CNA in Arabidopsis
Source: PLoS One. 2015 May 26;10(5):e0126006. doi: 10.1371/journal.pone.0126006 (PMC4444308; doi:10.1371/journal.pone.0126006)
Supplement: S1 File — Table A. Markers used for map-based cloning of ago10-15 from Fig 1A.Table B. Primers used for WUS and CLV3 transcript analysis. Fig A. ago10-15 allelism test. Fig B. wus suppression requires phb, phv and cna homozygousity. Fig C. WOX5 transcript accumulation unchanged in phb phv cna mutants. (DOCX) [file pone.0126006.s001.docx]

**Supporting Information**

**Supplemental Table A.**  Markers used for map-based cloning of *ago10-15* from Figure 1A.

|  | Marker | Enzyme | Forward | Reverse | Col | Ler |
| --- | --- | --- | --- | --- | --- | --- |
| A | MNL12- lch1 |  | AGCCGCAAATGGATGTCTCACCA | TGACCCCTCCACATCTCCCAACT | 317 | 303 |
| B | K9D7-lch2 |  | TGTTTGCAGTGATTGGTGGT | TCCCCTTTACCTTCACATGC | 407 | 392 |
| C | MQD19-lch3 |  | GGCCAAATTTTCAACGACAA | TTGGGGAGAGTTTGTGTGGT | 663 | 606 |
| D | MRH10-lch2 (CAPS) | DdeI | CGCAAGCCCATTACAAAAAT | AAGGATCTGCTTTGCTTGGA | 249 | 353 |
| E | MFC16-lch1 |  | GGGCGGACTTGTAAAACTT | CGACAATTTTGGGAGCAGAT | 332 | 322 |
| F | K23L20-lch1 |  | GATTTCGCTCTCTGCCAAAA | CGACCGTTTGCTATGCTTCT | 156 | 90 |
| G | K9E15-lch1 |  | CGCGGAATGAAACTACCACT | TCCTCAACTGATGTGCTTGC | 187 | 168 |

**Supplemental Table B**. Primers used for *WUS* and *CLV3* transcript analysis

| RT-PCR Primers | Forward | Reverse |
| --- | --- | --- |
| WUS^1^ | ACAAGCCATATCCCAGCTTCA | CCACCGTTGATGTGATCTTCA |
| CLV3^2^ | GCTACTACTACTACTCTTCTGCTTCTTGTT | GCTGTCTTGGTGGGTTCACA |
| TUB | AGAGGTTGACGAGCAGATGA | CCTCTTCTTCCTCCTCGTAC |
| WOX5-primer1 | CGGCAAGATAGAGAGCAAGAA | GATCTAATGGCGGTGGATGT |
| WOX5-primer2 | CGGTGGAGCAGTTGAAGATA | CACCTTGGAGTTGGAGTCTT |

WUS^1^ : WUS primers were used as described [[1](#_ENREF_1)].

CLV3^2^ : CLV3 primers were used as described [[2](#_ENREF_2)].

1. Kwon CS, Chen C, Wagner D (2005) WUSCHEL is a primary target for transcriptional regulation by SPLAYED in dynamic control of stem cell fate in Arabidopsis. Genes Dev 19: 992-1003.

2. Muller R, Borghi L, Kwiatkowska D, Laufs P, Simon R (2006) Dynamic and compensatory responses of Arabidopsis shoot and floral meristems to CLV3 signaling. Plant Cell 18: 1188-1198.


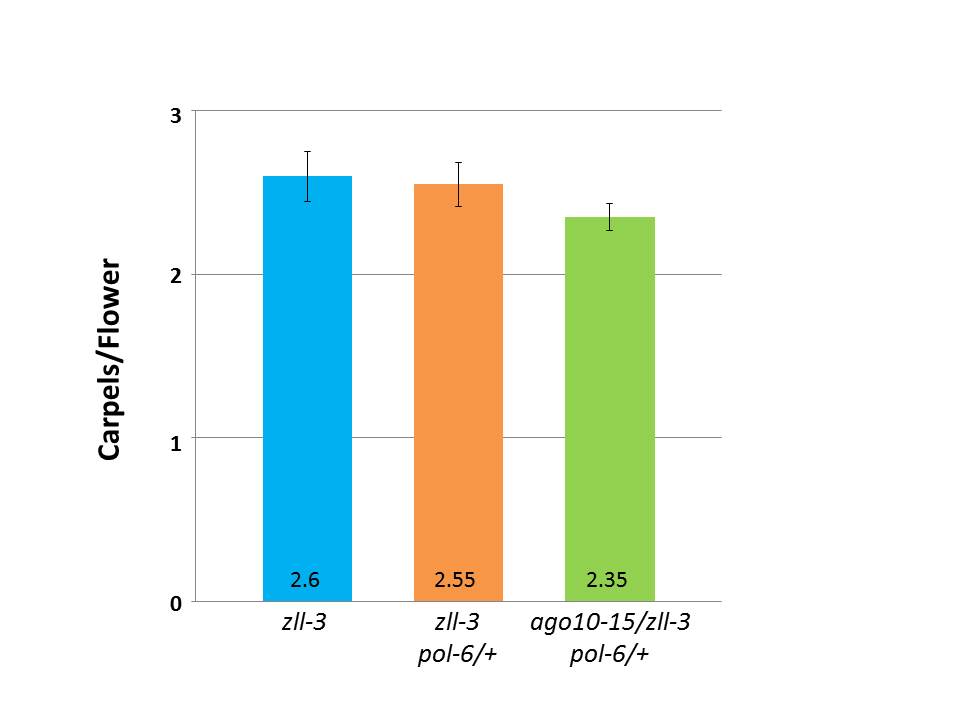
Supplemental Figure A. *ago10-15* allelism test.

Supplemental Figure A. *ago10-15* allelism test.

Mean number of carpels per flower with standard error of the mean for an allelism test between *ago10^zll-3^* and *ago10-15*. F1 progeny of *ago10^zll-3^* x *pol-6* and *ago10^zll-3^* x *ago10-15 pol-6* were assessed for phenotype.

Supplemental Figure B. *wus* suppression requires *phb*, *phv* and *cna* homozygousity.


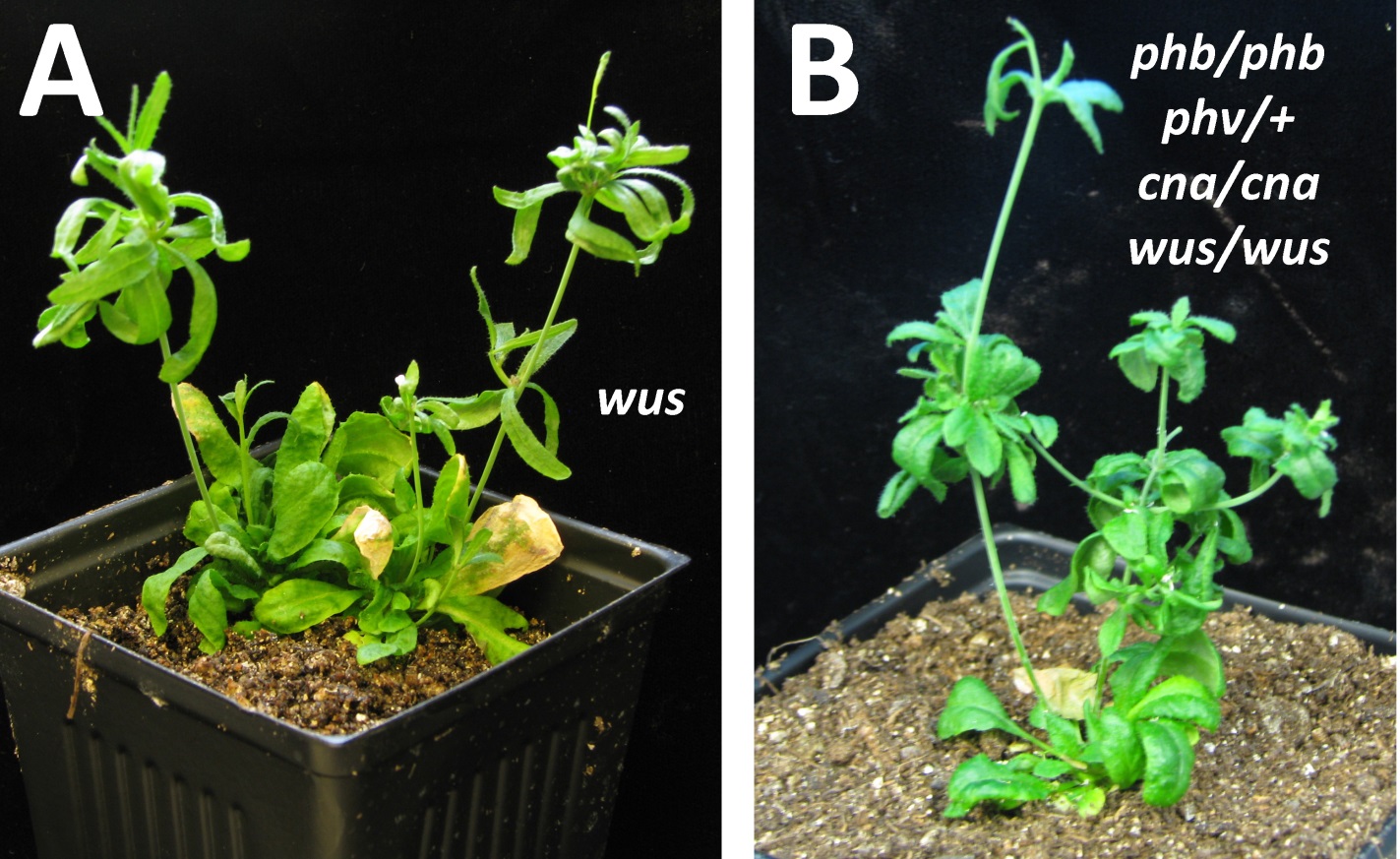


Supplemental Figure B. *wus* suppression requires *phb*, *phv* and *cna* homozygosity.

Mature *wus-1*/*wus-1* and *wus-1*/*wus-1* *phb*/*phb* *phv*/+ *cna*/*cna* plants are shown.


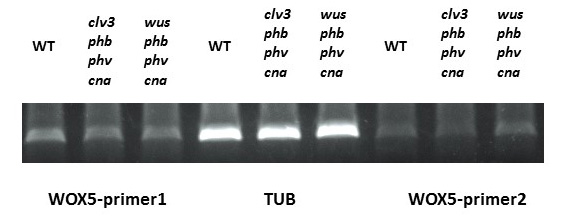
Supplemental Figure C. *WOX5* transcript accumulation unchanged in *phb phv cna* mutants.

Supplemental Figure C. *WOX5* transcript accumulation unchanged in *phb phv cna* mutants. Semi-quantitative RT-PCR measuring *WOX5* and control *TUBULIN* transcripts in wild-type (WT), *clv3 phb phv cna*, and *wus phv phb cna* 14-days-old seedlings. See Supplemental Table B for the sequence of primers.
